# Post-Marketing Nutrivigilance of Red Yeast Rice-Containing Food Supplements: Spontaneous Adverse Event Reports and Exposure-Based Reporting Rate Estimates

**DOI:** 10.3390/nu18142302

**Published:** 2026-07-14

**Authors:** Arrigo Francesco Giuseppe Cicero, Federica Fogacci

**Affiliations:** 1 Hypertension and Cardiovascular Risk Research Center, Medical and Surgical Sciences Department, Alma Mater Studiorum University of Bologna, 40130 Bologna, Italy; federica.fogacci@studio.unibo.it; 2 Cardiovascular Medicine Unit, Heart, Chest and Vascular Department, IRCCS Azienda Ospedaliero-Universitaria di Bologna, 40138 Bologna, Italy; 3 Department of Medical Pharmacology, Medical Faculty, Ataturk University, 25240 Erzurum, Turkey; 4 Italian Society of Nutraceuticals (SINut), 40100 Bologna, Italy

**Keywords:** red yeast rice, nutrivigilance, food supplements, adverse events, post-marketing surveillance, hypercholesterolemia, safety profile, spontaneous reporting, causality assessment, phytovigilance

## Abstract

**Background and Objectives:** Red yeast rice (RYR)-containing food supplements are widely used for mild-to-moderate hypercholesterolemia, but their post-marketing safety remains relevant. This study characterized their safety profile within a structured multicompany nutrivigilance framework. **Methods:** Spontaneous reports of suspected adverse events (AEs) associated with RYR-containing food supplements were collected between 12 December 2022 and 31 May 2024. Cases were documented using a structured case report form, medically reviewed, and classified for causality according to the World Health Organization–Uppsala Monitoring Centre framework. Exposure was estimated from cumulative manufacturing and sales data. **Results:** A total of 148 AE reports were collected from 12 European countries. Estimated exposure corresponded to 608,815 exposed subject-periods over 18 months, based on cumulative manufacturing and sales data and recommended daily dosage. Mean age was 65.3 ± 13.3 years; among reports with available age, 58.1% occurred in individuals aged ≥70 years. Gastrointestinal events were most frequent (45.9%), followed by arthromyalgia (16.9%) and hypersensitivity reactions (10.8%). Most events were mild or moderate (97.9%); severe and serious events accounted for 0.7% and 1.4%, respectively. Both serious AEs occurred in individuals aged ≥70 years. The estimated exposure-based reporting rate was 0.02% for all AEs and 0.0003% for serious AEs. **Conclusions:** Within a structured post-marketing nutrivigilance framework, RYR-containing food supplements showed a low reporting frequency of AEs relative to estimated exposure, with most events being mild or moderate. Future research should strengthen harmonized nutrivigilance systems and integrate product-specific exposure data, formulation characteristics, and post-marketing safety monitoring to support more precise risk assessment.

## 1. Introduction

Cardiovascular disease remains the leading cause of morbidity and mortality worldwide, and dyslipidemia continues to rank among its most important modifiable determinants [1,2]. Yet the contemporary burden of atherosclerotic cardiovascular disease (ASCVD) is not driven only by the small fraction of individuals at very high risk [3]. It also reflects the much larger number of people who spend decades with mildly abnormal but persistent cardiometabolic risk-factor levels [4]. In this sense, the challenge of prevention is not confined to identifying advanced risk; it also concerns the capacity to preserve favorable risk-factor profiles before disease pathways become firmly established. This is the essence of primordial prevention [5], namely, maintaining blood pressure, low-density lipoprotein cholesterol (LDL-C), adiposity, and glycaemia within healthy ranges before progression toward overt disease [5,6]. The relevance of this perspective has become even clearer as the global prevalence of potentially preventable cardiometabolic risk factors continues to rise across populations [7,8].

This perspective is closely aligned with Geoffrey Rose’s population principle: a large number of people exposed to a small excess risk may generate more disease burden than a small number exposed to high risk [9]. The practical implication is that the greatest gains in cardiovascular prevention may come not only from targeting the highest-risk individuals, but also from gently shifting entire population distributions toward healthier ranges [10]. For LDL-C, blood pressure, glycaemia, and adiposity, even modest average downward shifts can translate into meaningful reductions in incident disease when applied across broad population groups. Such a framework is particularly relevant in Europe, where lifestyle interventions are of fundamental importance, pharmacotherapy is not routinely indicated in many low-risk individuals, and the challenge is often not only treatment intensity but timing, feasibility, and adherence across the life course [8,11].

Among cardiometabolic risk factors, LDL-C retains a central role because its association with ASCVD is causal, cumulative, and strongly time-dependent [12,13]. This makes LDL-C lowering one of the core objectives of cardiovascular prevention [14,15]. Importantly, contemporary evidence supports the view that earlier and more sustained reduction in LDL-C exposure may yield substantially greater proportional reductions in lifetime cardiovascular risk than interventions introduced later in life [16,17]. This concept is especially relevant in primary prevention, where many individuals remain below formal treatment thresholds despite persistent suboptimal lipid levels and prolonged biological exposure to atherogenic lipoproteins [7,14]. In such individuals, the question is not whether risk is already extreme, but whether the trajectory of risk can be modified before it becomes so [7,18].

Lifestyle interventions are of fundamental importance in the management of mild-to-moderate hypercholesterolemia, particularly in individuals at low-to-moderate global cardiovascular risk [19]. Dietary modification, physical activity, weight control, and smoking avoidance remain indispensable [20,21,22,23,24,25]. However, even under favorable conditions, the lipid-lowering effect of lifestyle measures alone is often modest, and long-term adherence is variable [26,27]. Dietary and nutraceutical strategies should therefore be interpreted through two complementary perspectives. At the individual level, the absolute clinical benefit of LDL-C lowering may be greater in patients with higher baseline LDL-C or higher global cardiovascular risk; however, these are also the patients in whom guideline-directed lipid-lowering pharmacotherapy should not be delayed, replaced, or diluted by non-pharmacological approaches alone [14,15]. At the population level, by contrast, modest but sustained improvements in LDL-C among many low-to-moderate-risk individuals may still be clinically meaningful because they reduce cumulative exposure to atherogenic lipoproteins before pharmacological treatment thresholds are reached [8,16,17,28]. This distinction reconciles individual-level efficacy with a Rose-inspired population-prevention perspective: nutraceuticals are not proposed as more potent substitutes for drugs in high-risk patients, but as standardized, evidence-based adjuncts that may support healthier risk-factor trajectories in appropriately selected individuals [8,18,26,28]. In this setting, adjunctive and evidence-based nutraceutical strategies have attracted increasing attention, particularly for individuals who do not qualify for pharmacologic therapy, are unwilling to initiate statins, or seek additional non-pharmacological LDL-C reduction within a broader preventive framework [8,18,26,29,30,31].

Among lipid-lowering nutraceuticals, red yeast rice (RYR) occupies a distinctive position [32,33]. Produced by the fermentation of rice, usually by *Monascus purpureus*, RYR contains several monacolins, among which monacolin K acts through reversible inhibition of 3-hydroxy-3-methylglutaryl coenzyme A reductase [33,34]. This mechanism largely explains why RYR has repeatedly shown clinically meaningful lipid-lowering effects and why its use has long attracted the attention of both lipidologists and regulators [35,36,37]. In contrast to many other supplements, the efficacy of RYR has been documented in randomized trials, systematic reviews, and narrative reviews, with LDL-C reductions versus placebo broadly ranging from approximately 15% to 34% [18,38,39].

This body of evidence has supported the view that RYR may have a role in selected patients with mild-to-moderate hypercholesterolemia, especially when the objective is to strengthen lifestyle-based prevention rather than substitute for clearly indicated pharmacologic treatment [40]. The potential contribution of RYR-containing nutraceuticals should therefore be interpreted within a graded preventive model: not as an alternative to statins in patients who clearly require pharmacologic lipid lowering, but as a possible adjunct in lower-risk individuals whose LDL-C remains suboptimal despite non-pharmacologic measures or in those unwilling to initiate drug treatment [41,42]. Framed in this way, RYR becomes relevant not because it solves the problem of dyslipidemia universally, but because it may contribute to a “shift-the-curve” strategy in populations whose absolute risk is individually modest but collectively consequential [8,18,26,43].

Recent interventional data have further strengthened this rationale, particularly for contemporary formulations containing lower doses of monacolins and additional nutraceutical components. A double-blind, placebo-controlled randomized clinical trial showed that a formulation combining RYR with fish oil standardized in polyunsaturated fatty acids was well tolerated and associated with significant improvements in LDL-C, total cholesterol, apolipoprotein B, high-sensitivity C-reactive protein, and endothelial reactivity after 8 weeks of supplementation [44]. Likewise, a PRISMA-compliant systematic review and meta-analysis of randomized controlled clinical trials evaluating Armolipid Plus^®^ reported significant improvements in LDL-C, total cholesterol, triglycerides, hsCRP, and fasting glucose, together with an overall favorable tolerability profile [45]. These observations suggest that the clinical discussion surrounding RYR can no longer be reduced to whether it works; the more relevant questions concern formulation, dose, patient selection, safety context, and post-marketing monitoring.

At the same time, the safety assessment of RYR remains more complex than efficacy alone. Because monacolin K is structurally identical to lovastatin, RYR lies at the interface between food supplementation and statin-like pharmacology [46]. As with many food supplements, commercial preparations may differ in composition, monacolin content, and manufacturing characteristics [47]. These differences highlight the importance of product standardization, quality assurance, and transparent labelling to support consistent consumer use and interpretation of safety data. More broadly, the interpretation of tolerability data is complicated by differences across marketed products, possible contamination, labeling variability, and the likelihood of concomitant exposure to other supplements or drugs in real-world use. These factors make it difficult to extrapolate uniformly from randomized trial data to all marketed formulations and underscore the importance of formulation-specific safety evaluation [18,38,48].

The European regulatory context has made this issue even more salient. Following the 2018 EFSA Scientific Opinion on the safety of monacolins from RYR, the European Commission adopted Regulation (EU) 2022/860, which restricted the daily intake of monacolins from RYR in food supplements to below 3 mg and introduced additional warning requirements [49]. More recently, the 2025 EFSA NDA Scientific Opinion reviewed additional data submitted during the Union scrutiny period, including analytical data, nutrivigilance and post-marketing evidence, case reports, and clinical studies, and concluded that the available evidence still did not allow establishing the safety of monacolins in RYR supplements below 3 mg/day or identifying a daily intake that does not raise safety concerns for the general population or vulnerable subgroups [37]. This regulatory shift did not arise from lack of evidence that RYR lowers LDL-C, but from continuing uncertainty regarding the extent to which available data were sufficient to define a safe exposure threshold across heterogeneous products, consumer groups, and patterns of use. In this sense, the central question is no longer simply whether RYR is effective, but whether its real-world use can be monitored with enough methodological rigor to support a clinically meaningful safety profile [36].

This tension is also reflected in the evolving guideline landscape. The 2025 Focused Update of the ESC/EAS dyslipidemia guidelines states that food supplements or vitamins without documented safety and significant LDL-C-lowering efficacy are not recommended to lower ASCVD risk [50]. At the same time, that statement does not amount to a categorical dismissal of all lipid-lowering nutraceuticals. Rather, it reinforces a stricter standard: only products supported by credible efficacy data, acceptable safety documentation, and sufficient standardization should be considered. In this reading, the role of nutraceuticals is not weakened but clarified. They belong, if anywhere, in a disciplined preventive strategy aimed at supporting healthy trajectories in appropriately selected individuals, especially when the goal is to reduce long-term exposure to suboptimal cardiometabolic risk factors rather than replace proven therapies in high-risk settings [8,43,50].

From a methodological perspective, randomized controlled trials alone cannot fully resolve this issue. Clinical trials are essential to establish efficacy and short-term tolerability of food supplements, but they are usually conducted in selected populations, over limited durations, and under conditions that may not capture rare, delayed, or context-dependent adverse events. This limitation is particularly relevant for food supplements, which are often consumed outside specialist supervision and sometimes in combination with other products or medications. In such a context, post-marketing surveillance is not ancillary but necessary [51,52]. Yet Europe still lacks a harmonized, standardized nutrivigilance system for food supplements, and this fragmentation limits the ability to interpret spontaneous adverse-event data consistently across products and countries [53].

This gap is especially important for RYR. Recent post-marketing nutrivigilance evidence involving a widely used RYR-containing product line has highlighted low overall reporting rates relative to exposure while also underscoring that meaningful safety assessment depends on structured surveillance, product quality, and careful contextualization of case reports. Such findings are reassuring, but they do not eliminate the need for additional real-world evidence generated within transparent and methodologically explicit surveillance frameworks. On the contrary, they strengthen the case for studies capable of combining standardized case collection, expert medical review, causality assessment, and exposure-based denominators, thereby moving spontaneous reporting data beyond anecdotal signal generation toward clinically interpretable risk characterization [53].

Against this background, the present study was designed to characterize the post-marketing safety profile of RYR-containing food supplements within a structured nutrivigilance framework. Specifically, the aim was to describe the pattern of spontaneously reported suspected adverse events, assess their causality and severity, explore selected subgroup distributions, and contextualize reported events through exposure-based reporting rate estimates. By integrating case-level clinical evaluation with cumulative exposure data, this study seeks to provide a clinically meaningful real-world perspective on the safety of RYR-containing food supplements in contemporary European practice and to inform a more responsible, evidence-based use of food supplements in cardiovascular prevention.

## 2. Methods

### 2.1. Study Design and Surveillance Framework

**Figure 1:**
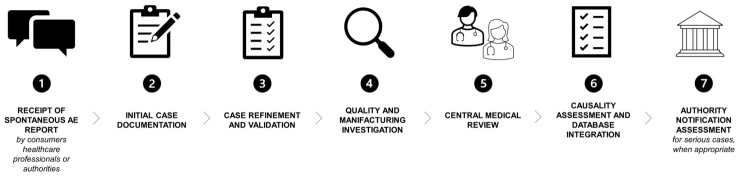
Workflow of the post-marketing nutrivigilance system for red yeast rice-containing food supplements. The diagram summarizes the sequential steps of the surveillance process, including receipt of spontaneous reports, initial documentation, case refinement and validation, quality and manufacturing investigation when appropriate, central medical review, causality assessment, database migration, and authority notification for serious cases when applicable.

This study presents the findings from a pilot post-marketing nutrivigilance initiative focused on food supplements containing RYR, conducted within the framework of the EHPM alliance and coordinated in collaboration with the Medical and Surgical Sciences Department (DIMEC) of the University of Bologna. The initiative was conceived as a multicompany surveillance program designed to collect spontaneous reports of suspected adverse events (AEs) arising during the use of RYR-containing supplements over an 18-month observation period, from 12 December 2022 to 31 May 2024. In this nutrivigilance context, the term “adverse events” refers to spontaneously reported, unexpected or undesirable events temporally associated with product use, without implying a confirmed causal relationship or a pharmaceutical classification of the product. Reports could be submitted by consumers, healthcare professionals, including physicians, pharmacists, and nutritionists, as well as health or regulatory authorities. Participating companies implemented a shared nutrivigilance methodology; companies with established internal surveillance procedures were permitted to maintain their own processes, provided that case-level data were subsequently transmitted to the project coordinator in a standardized format. The overall surveillance workflow is summarized in Figure 1.

### 2.2. Case Ascertainment, Case Report Form, and Data Collection

Each participating company designated a Nutrivigilance Officer (NV Officer) responsible for documenting and managing any spontaneously reported AE received through routine communication channels, including telephone, e-mail, social media, and direct consumer contact. To harmonize case collection across participating companies, data were recorded in a structured case report form (CRF) specifically developed for the project, while companies with pre-existing nutrivigilance systems were allowed to retain their internal procedures provided that case-level information was subsequently transferred in a standardized format. The CRF was designed to capture the minimum information required for clinical evaluation and causality assessment, including end-user characteristics, suspect product identity and lot information, indication and modality of use, timing and outcome of the event, narrative case description, relevant medical history and concomitant products, dechallenge/rechallenge information, seriousness criteria, reporter qualification, and permission for follow-up contact. The full CRF is provided in Supplementary Materials. The form was intended to ensure consistency of case capture across participating companies while preserving compatibility with pre-existing internal nutrivigilance procedures. Active follow-up was specifically undertaken whenever core case elements were missing, incomplete, or internally inconsistent, including product identification, lot number, medical documentation in serious cases, end-user characteristics, event description and outcome, indication for use, and dates or modalities of product exposure and event onset. Potential duplicate reports were identified through comparison of end-user characteristics, event description and occurrence date, and suspect product information, and were reconciled before inclusion in the analytical dataset. In serious cases for which lot information was available, a product quality or manufacturing investigation was requested and performed when appropriate. Completed documentation, together with supporting materials such as photographs, product carton artwork, and certificates of analysis when available, was then submitted for expert medical review (Figure 1).

### 2.3. Medical Review and Causality Assessment

Anonymized case reports were centrally reviewed by medical experts. Case evaluation included assessment of event severity, review of product-related factors, comparison with previously reported cases, and appraisal of the likelihood of a causal relationship between the AE and the suspect product. Causality was classified according to the World Health Organization (WHO)–Uppsala Monitoring Centre (WHO-UMC) framework [54]. For serious cases, notification to the relevant national competent authority was considered by the reporting company, where appropriate. All reports were therefore subjected to expert medical review and causality assessment before inclusion in the cumulative safety evaluation.

### 2.4. Exposure Estimation and Statistical Analysis

The CRF captured a broader set of case-level variables than those included in the present analysis. For the purposes of this study, the principal variables of interest were country of occurrence, age, sex, time to AE onset, AE category, causality assessment, and severity grading. Time to onset was categorized as <1 week, <1 month, >1 month, or unknown. AE categories included gastrointestinal events, arthromyalgic events, hypersensitivity reactions, blood laboratory alterations, urogenital events, cardiovascular events, central nervous system and sensorium events, and other events. Continuous variables were summarized as mean ± standard deviation, whereas categorical variables were expressed as counts and percentages. Exploratory subgroup analyses by sex and age group (<70 vs. ≥70 years) were conducted using the chi-square test. These subgroup comparisons were considered exploratory in nature and were restricted to reports with available data only, given variable completeness across CRF fields. Product-level information on RYR content and monacolin K content was available for the suspected products included in the nutrivigilance dataset. The exact monacolin K content varied across products; however, all included products were formulated to provide less than 3 mg/day of monacolin K according to the recommended daily dose, in line with the current European regulatory framework. The present analysis was therefore designed to characterize the aggregate post-marketing safety profile of RYR-containing food supplements marketed under this regulatory threshold, rather than to correlate adverse-event reporting with small between-product differences in monacolin K content. Accordingly, formal dose–response analyses according to exact monacolin K exposure, as well as product-specific comparisons across formulations, were not performed. Therefore, the analysis was conducted at the level of the food supplement category captured by the nutrivigilance initiative. Consumer exposure over the study period was estimated from cumulative manufacturing and sales data provided by the companies that shared market data for the RYR-containing food supplements included in the present nutrivigilance analysis. These included Viatris, Tilman, Perrigo, Unifarco, Naturando, Difass International S.p.A., PIAM Farmaceutici S.p.A., PharmaExtracta, and CSO. Although product names are not reported for confidentiality reasons, these aggregate data were converted into exposed subject-periods based on the recommended daily dosage, allowing calculation of exposure-based reporting rate estimates. Accordingly, the denominator used for reporting rate estimation reflects total estimated consumer exposure derived from market data, rather than the number of individuals actively monitored within the surveillance system. This approach provided a denominator-based contextualization of spontaneous reports, which are otherwise difficult to interpret quantitatively. However, because exposure was not directly observed at the individual level, these estimates should be regarded as approximate exposure-based reporting rates rather than true epidemiological incidence rates. The validity of this approach is therefore limited to providing an order-of-magnitude estimate of reporting frequency relative to projected market exposure, not to determining individual-level risk.

Cumulative sales data were converted into exposed subject-periods assuming continuous use at the recommended daily dose under a 100% compliance assumption over the entire 18-month study period. This estimate should be regarded as a full-adherence exposure equivalent rather than a directly observed number of exposed individuals. Because real-world use of food supplements is often intermittent, the same cumulative number of sold units may correspond to a larger number of consumers exposed for shorter or less regular periods. To account for this uncertainty, a hypothetical 50% average compliance scenario was also considered. Under this scenario, the same number of sold units would correspond to twice the number of exposed subject-periods compared with the full-adherence assumption. The 100% and 50% compliance scenarios were therefore used to provide a plausible range of exposure-based reporting rates. These estimates should be interpreted as approximate reporting rates relative to projected market exposure, not as true epidemiological incidence rates or measured estimates of individual compliance.

## 3. Results

### 3.1. Reporting and Demographic Characteristics

**Figure 2:**
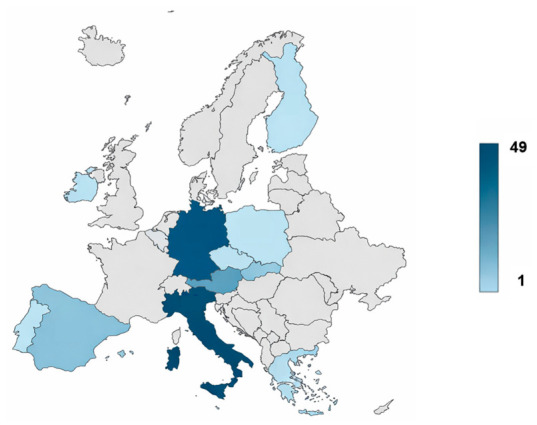
Geographic distribution of spontaneous adverse event reports related to red yeast rice-containing food supplements across participating European countries. The map shows the absolute number of spontaneous reports by country. It does not represent country-specific incidence or reporting rates, because country-level sales or exposure denominators were not available. Therefore, between-country differences may reflect differences in market size, product use, reporting behavior, or national reporting practices, and should not be interpreted as evidence of different safety risks across countries.

A total of 148 spontaneous reports of suspected adverse events (AEs) associated with RYR-containing food supplements were collected between 12 December 2022 and 31 May 2024. Reports originated from 12 European countries, with the highest numbers recorded in Italy (*n* = 49), Germany (*n* = 34), Austria (*n* = 21), and Spain (*n* = 19), whereas the remaining reports were distributed across Slovakia, Belgium, Greece, Portugal, Poland, the Czech Republic, Finland, and Ireland (Figure 2). These country-level counts represent absolute numbers of spontaneous reports and were not normalized by country-level sales or exposure denominators, because such denominators were not available in a sufficiently complete and comparable format. Accordingly, between-country differences should be interpreted as differences in reporting counts rather than as differences in country-specific incidence or risk.

Age information was available for 93 reports; the mean age was 65.3 ± 13.3 years, and 54 (58.1%) occurred in individuals aged ≥70 years. Sex information was available for 145 reports; 112 (77.2%) occurred in women and 33 (22.8%) in men. This marked female predominance describes the distribution of spontaneous reports with available sex information and should not be interpreted as evidence of a higher sex-specific risk, because sex-stratified exposure denominators were not available. These reporting and demographic characteristics are summarized in Table 1.

### 3.2. Temporal Profile and Clinical Distribution of Reported Adverse Events

**Figure 3:**
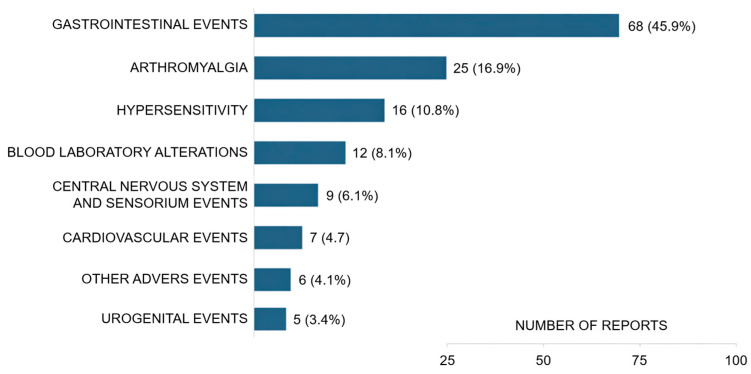
Distribution of reported adverse event categories associated with red yeast rice-containing food supplements. Horizontal bar chart showing the frequency and proportion of the main adverse event categories reported during the study period. Each bar reports both the absolute number of spontaneous reports and the corresponding percentage of the total number of reports. Gastrointestinal events were the most frequently reported category, followed by arthromyalgia and hypersensitivity reactions. Categories represent reported clinical event groups and do not imply confirmed causality. Percentages were calculated on the total number of spontaneous reports.

Information on time to onset was unavailable for 116 reports (78.4%). Therefore, only 32 reports were evaluable for temporal analysis, and the corresponding onset distribution should be interpreted with substantial caution. Among the 32 reports with available timing data, 18 events (56.3%) occurred within 1 week of product use, 5 (15.6%) within 1 month, and 9 (28.1%) after more than 1 month. Because of the high proportion of missing time-to-onset information, these proportions should be regarded as descriptive findings limited to reports with available timing data and should not be extrapolated to all spontaneous reports or used to infer a reliable temporal risk pattern. Gastrointestinal events were the most frequently reported AE category (*n* = 68, 45.9%), followed by arthromyalgia (*n* = 25, 16.9%) and hypersensitivity reactions (*n* = 16, 10.8%). Together, these three categories accounted for nearly three-quarters of all reported events (73.6%). Less frequently reported categories included blood laboratory alterations, central nervous system and sensorium events, cardiovascular events, other events, and urogenital events. The distribution of AE categories is shown in Figure 3.

### 3.3. Causality Assessment and Severity

According to the WHO-UMC causality classification, 17 reports (11.5%) were classified as certain, 7 (4.7%) as probable/likely, 88 (59.5%) as possible, 26 (17.6%) as unlikely, and 10 (6.8%) as unclassified (Table 2). Overall, 24 reports (16.2%) were categorized as certain or probable/likely. When causality and clinical seriousness were considered together, no serious adverse event was classified as certain or probable/likely. The two serious reports were both assessed as unlikely according to the WHO-UMC causality criteria. The severity profile was predominantly mild to moderate: 126 reports (85.1%) were graded as mild and 19 (12.8%) as moderate, whereas only 1 report (0.7%) was graded as severe and 2 reports (1.4%) as serious. The two serious adverse events (SAEs), both reported in Germany by the affected individuals, concerned bladder congestion leading to hospitalization in a 71-year-old woman and pancreatitis in a 74-year-old man. Both cases were spontaneous reports and lacked medical confirmation. This classification indicates that, based on the available case information, a causal relationship with the suspect product was considered improbable rather than established or likely. In both serious reports, the available documentation was insufficient to support a consistent causal association, and no medical confirmation was available. Therefore, these cases should be interpreted as serious spontaneous reports temporally associated with product use, but not as serious adverse reactions causally attributed to RYR-containing food supplements. In both reports, use of the food supplement was considered improper because the patients were older than the recommended age limit of 70 years; moreover, in the pancreatitis case, the daily amount of food supplement consumed was not specified in the corresponding nutrivigilance report form. Overall, severe or serious events were uncommon, accounting for 3 of 148 reports (2.0%).

### 3.4. Exploratory Subgroup Analyses

Sex-stratified analyses showed a significant difference in the distribution of onset categories between women and men (*p* = 0.018), although this result should be interpreted cautiously in light of the substantial proportion of reports with missing onset information. No significant sex-related differences were observed for AE category (*p* = 0.160), causality classification (*p* = 0.134), or severity grading (*p* = 0.190). Likewise, no statistically significant differences emerged across age groups (<70 vs. ≥70 years) for onset category, AE type, causality classification, or severity. Both SAEs occurred in individuals aged ≥70 years. Taken together, these subgroup findings should be considered exploratory.

### 3.5. Exposure-Based Reporting Rate Estimates

Based on cumulative manufacturing and sales data available up to 31 May 2024, more than 328,759,726 tablets containing RYR were sold during the study period. Based on the recommended daily dosage, and under the idealized assumption of 100% adherence throughout the study period, this corresponded to an estimated 608,815 exposed subject-periods over 18 months. This exposure estimate was used as the denominator for reporting rate calculations and reflects projected consumer exposure derived from market data, rather than a defined cohort of actively monitored individuals. The exposure denominator was available only at the aggregate level and was therefore suitable for estimating the overall reporting rate, but not for calculating country-specific reporting rates. Because sales data were not available from all participating companies, this denominator is likely to underestimate the actual consumer exposure to the RYR-containing food supplements captured by the surveillance initiative. Therefore, the estimated exposure-based reporting rates of reported AEs and serious AEs, 0.02% and 0.0003%, respectively, should be interpreted as largely conservative with respect to the exposure denominator, as they are likely to overestimate rather than underestimate the reporting rate (Table 3). As a sensitivity analysis, we also calculated a second hypothetical scenario assuming 50% compliance, in which the estimated exposure denominator increased from 608,815 to 1,217,630 exposed subject periods. Under this scenario, the estimated reporting rates would be 0.012% for all reported AEs and 0.00016% for serious AEs (Table 3). Together, the 100% and 50% compliance scenarios provide a plausible range of exposure-based reporting rates, acknowledging that real-world use is unlikely to correspond exactly to either extreme.

## 4. Discussion

The present study provides a structured post-marketing assessment of the safety profile of RYR-containing food supplements in contemporary European practice. Overall, within the limits of spontaneous reporting and exposure-based estimation, the findings support a reassuring tolerability profile: most reported adverse events were mild or moderate, severe and serious cases were rare, and the estimated reporting rate remained low when interpreted against cumulative exposure. Importantly, this estimated rate should be regarded as conservative. The exposure denominator was based on available sales data converted into full-adherence 18-month exposure equivalents, assuming continuous intake at the recommended daily dose. Since real-world use of food supplements is commonly intermittent, the same number of sold units probably reflects a larger number of individual consumers exposed for shorter or less regular periods. Together with the incomplete availability of company sales data, this implies that the calculated AE reporting rate is more likely to overestimate than underestimate the true reporting frequency. In that sense, the study adds clinically meaningful real-world information to a field in which efficacy has often been discussed more extensively than post-marketing safety.

This point matters because the relevance of RYR is not confined to its lipid-lowering effect. Its broader significance lies in the possibility that a well-characterized nutraceutical may contribute to prevention before overt disease pathways become firmly established. That is the logic of primordial prevention: keeping risk-factor levels favorable before pharmacologic treatment becomes necessary. It is also entirely consistent with Rose’s population principle, according to which a large number of people exposed to a small excess risk may generate more disease burden than a small number exposed to high risk [9]. In practical terms, the potential value of interventions such as evidence-based nutraceuticals may therefore lie not only in their effects on selected individuals, but also in their capacity to help shift population distributions of LDL-C, blood pressure, adiposity, and glycaemia toward healthier ranges over time [7,8].

Within this framework, the current findings are especially relevant because the clinical efficacy of RYR has already been supported by substantial literature [18,38,40,44,45,55,56,57]. The present study complements that efficacy literature by showing that, in routine use and under a structured surveillance system, reported adverse events were infrequent relative to estimated exposure and were predominantly non-severe.

An additional finding deserves particular attention. Among reports with available age information, individuals aged ≥70 years accounted for more than half of cases, and both serious adverse events occurred in this subgroup. This observation is clinically relevant because individuals older than 70 years are explicitly recognized in the current European regulatory framework as a group for whom the use of RYR-containing food supplements is not recommended [49]. Because stratified exposure data by age were not available, a formal calculation of age-specific incidence or relative risk for individuals aged ≥70 years was not possible; therefore, the present dataset cannot establish that risk is intrinsically higher in older users. Nevertheless, the occurrence of both SAEs in patients above the recommended age limit suggests that the most clinically relevant reports in this series may be related, at least in part, to use outside labelled conditions of use and warnings, rather than to a generalized safety signal across all exposed consumers. The possible contribution of age-related comorbidities and concomitant medications cannot be excluded, although it could not be formally assessed in the present dataset. Importantly, both serious reports were classified as unlikely according to the WHO-UMC causality framework. Thus, although they were clinically relevant because they led to hospitalization and occurred in individuals older than the recommended age limit, they should not be interpreted as confirmed or likely product-related serious adverse reactions. This distinction is essential for regulatory interpretation, because seriousness and causality are independent dimensions in post-marketing safety assessment. At the same time, the spontaneous reporting of both SAEs by the affected individuals suggests a meaningful level of consumer awareness and indicates that the surveillance framework was able to capture clinically relevant reports even in the absence of a confirmed causal relationship. Taken together, these observations shift part of the discussion from the intrinsic safety of the ingredient alone toward the clarity of consumer information, the visibility of warnings, and the practical effectiveness of guidance provided to users in real-world settings.

This interpretation is consistent with the current regulatory and guideline landscape. Following the 2018 EFSA Scientific Opinion on monacolins from RYR and the subsequent adoption of Regulation (EU) 2022/860, the European narrative moved away from efficacy alone and toward a more cautious position centered on uncertainty, vulnerable groups, and conditions of use [36,37]. More recently, the 2025 Focused Update of the ESC/EAS dyslipidemia guidelines stated that food supplements or vitamins without documented safety and significant LDL-C-lowering efficacy are not recommended to lower ASCVD risk, thereby reinforcing the principle that nutraceutical use must be evidence-based, formulation-specific, and clinically disciplined [43,50]. The present findings fit well within that framework: they do not support indiscriminate supplementation, but they do suggest that a substantial part of the observed safety burden may be linked to suboptimal adherence to labelled conditions of use and warnings, rather than to a generalized pattern of severe toxicity across all exposed consumers.

The predominance of gastrointestinal adverse events in the present series is noteworthy, but not unexpected. Gastrointestinal symptoms are among the most frequently described complaints in regulatory discussions and post-marketing reports concerning RYR. At the same time, the overall severity profile in the current study remained largely mild, which is clinically important. Severe or serious events were rare not only in absolute terms, but also when contextualized against estimated exposure. Thus, the reassuring interpretation of the present findings is based primarily on the internal structure of the dataset: the low number of spontaneous reports relative to estimated exposure, the predominance of mild or moderate events, the rarity of serious reports, and the systematic medical review and causality assessment applied to all cases. In that sense, the present data help distinguish between the existence of a reporting signal and the magnitude of clinically relevant real-world risk. This distinction is especially important in nutrivigilance, where spontaneous reports can easily appear more alarming than they are if interpreted without an exposure denominator or without adequate clinical review [53].

The relationship between RYR and statin tolerability also requires careful methodological framing. Although direct comparisons with statins should be interpreted cautiously because of major methodological differences in data sources, populations, exposure definitions, and reporting systems, the pattern of reported events in the present study shows some overlap with adverse reactions commonly described during statin therapy, particularly musculoskeletal symptoms [58]. This overlap is biologically plausible, given the statin-like pharmacology of monacolin K; however, available evidence in patients with statin-associated muscle symptoms has not consistently confirmed an increased incidence of myalgia with RYR use [59]. Moreover, a systematic review and meta-analysis of randomized controlled trials specifically evaluating RYR safety found no increased risk of musculoskeletal disorders compared with control, and no excess signal for non-musculoskeletal or serious adverse events [60]. Accordingly, the statin-related discussion should be interpreted only as supportive pharmacological context for understanding symptom patterns, not as evidence of equivalence between RYR-containing food supplements and statin safety data, nor as a basis for defining a safe intake threshold for monacolins. In the present post-marketing dataset, the estimated reporting frequency remained low when contextualized against projected consumer exposure, and serious reports were rare. These findings should therefore be interpreted as supportive real-world tolerability information within the methodological limits of spontaneous nutrivigilance, rather than as comparative safety evidence against medicinal-product datasets.

A further point of perspective may also be useful, particularly because RYR-containing products are positioned as food supplements rather than medicines. The following considerations are not intended to provide a direct epidemiological benchmark, to equate nutrivigilance-derived reporting rates with food-related adverse reactions, or to minimize safety considerations. Rather, they are intended as a qualitative contextual reference to avoid interpreting the mere occurrence of spontaneous reports as intrinsically exceptional. It is difficult to compare nutrivigilance-derived reporting rates directly with adverse reactions associated with conventional foods [61,62], because the underlying epidemiological metrics are fundamentally different. Nevertheless, adverse reactions to ordinary foods are not exceptional in the general population. In Europe, self-reported allergy to common foods has been reported to range broadly from 0.1% to 6.0%, and more recent challenge-confirmed estimates for specific foods such as cow’s milk are around 0.3% [63,64]. Moreover, food additives and excipients per se may also interact with the gut microbiota and contribute to gastrointestinal discomfort [62,65]. These figures cannot be used to infer relative risk or comparative safety for RYR-containing food supplements. They simply illustrate that undesirable reactions may occur even in relation to widely accepted and routinely consumed dietary exposures. For RYR-containing food supplements, the relevant safety interpretation depends instead on the frequency, severity, causality assessment, preventability, and appropriateness of use of reported events. In the present dataset, most reports were non-serious, serious cases were rare and assessed as unlikely, and the estimated reporting rate remained low even when uncertainty in the exposure denominator was explored through a conservative sensitivity scenario.

From a methodological perspective, one of the most relevant implications of the present study concerns the organization of nutrivigilance itself. The current experience shows that structured surveillance of suspected adverse events associated with RYR-containing supplements is feasible in routine practice. Standardized case collection, active follow-up when possible, duplicate assessment, medical review, causality classification, and exposure-based contextualization can all be implemented within a coherent framework. This is an important proof of concept, particularly in Europe, where a fully harmonized nutrivigilance infrastructure for food supplements is still lacking [53]. Importantly, the documentation of a measurable number of clinically plausible AEs should be interpreted not only as part of the safety profile under evaluation, but also as an indication that the surveillance framework was active and capable of capturing real-world events. By contrast, a complete absence of reports would have been more difficult to interpret, as it could have reflected limited system sensitivity rather than a genuine absence of AEs.

At the same time, feasibility does not necessarily mean optimal governance. In the long term, post-marketing surveillance of botanicals and food supplements should not rely primarily on manufacturers alone. Company-based systems can generate valuable data, but they inevitably risk procedural heterogeneity, variable case completeness, inconsistent follow-up, and lower perceived institutional independence. For products positioned at the interface between food supplementation and pharmacologically active compounds, a more appropriate model would be one in which manufacturers remain responsible for collecting and forwarding safety information, while signal detection, case standardization, cumulative evaluation, and public communication are anchored within an independent, government-coordinated surveillance infrastructure. The Italian phytovigilance experience provides an instructive model, with VigiErbe serving as an online platform for reporting suspected adverse reactions associated with food supplements, herbal products, and other natural products within a coordinated public surveillance system; in parallel, peer-reviewed analyses from the same framework have shown that institutional monitoring of natural products can generate clinically meaningful safety data over time [66].

The observed predominance of reports among women also warrants cautious interpretation. In the present dataset, women accounted for 112 of 145 reports with available sex information (77.2%). A similar female predominance has been described in other post-marketing datasets on RYR-containing products, including the recent EFSA assessment of nutrivigilance and post-marketing evidence. This pattern may reflect several non-mutually exclusive factors, including differences in supplement use, health-seeking behavior, awareness of symptoms, or propensity to report adverse events. However, because sex-stratified exposure data were not available, the present study cannot determine whether women had a higher reporting rate relative to exposure or a higher biological susceptibility to adverse events. Therefore, the observed sex distribution should be interpreted as a reporting pattern rather than as evidence of sex-specific risk.

The exploratory subgroup findings should be interpreted accordingly. The significant sex-related difference observed in onset distribution is difficult to interpret confidently because onset information was missing in a large proportion of reports. Likewise, the absence of statistically significant differences across age groups for onset category, adverse-event type, causality classification, or severity should not be over-read. In spontaneous-report datasets, subgroup analyses are often more useful for identifying patterns worthy of further investigation than for drawing robust comparative inferences. The value of the present subgroup findings therefore lies less in formal hypothesis testing than in highlighting clinically relevant contexts, most notably the concentration of the serious cases in individuals aged ≥70 years.

Several limitations should be acknowledged. First, the study is based on spontaneous reporting and is therefore intrinsically vulnerable to under-reporting, selective reporting, incomplete documentation, and variable reporting behavior across countries, companies, and reporters. However, under-reporting is expected to affect mainly non-serious events. SAEs are less likely to remain unreported once recognized by healthcare professionals or manufacturers, given applicable reporting obligations and the dedicated follow-up procedures used for serious cases in this surveillance framework. Therefore, while under-reporting cannot be entirely excluded, it is less likely to have substantially affected the ascertainment of SAEs. Second, some variables were incomplete in a substantial proportion of cases, particularly time to onset, which limits the robustness of temporal analyses. Indeed, time-to-onset information was missing in 116 of 148 reports (78.4%), leaving only 32 reports available for temporal evaluation. This missingness may reflect recall limitations, incomplete reporter information, or variability in follow-up across spontaneous reports, and it prevents any firm conclusion about the timing of adverse-event occurrence after product initiation. Accordingly, the observed distribution of onset categories should be interpreted only as descriptive information among reports with available timing data. Third, exposure was estimated indirectly from cumulative manufacturing and sales data rather than measured at the individual level, so the reporting-rate estimates should be interpreted as approximations rather than precise epidemiological rates. The exposure denominator was intended to contextualize spontaneous reports against projected market exposure, but it cannot substitute for a prospectively followed exposed cohort. Consequently, these estimates should be interpreted with substantial caution as exposure-based reporting rates, not as true incidence rates or direct measures of individual risk. In addition, sales data were not available from all participating companies; therefore, the exposure denominator is likely to be lower than the actual consumer exposure captured by the surveillance initiative. This makes the reporting-rate estimates conservative with respect to the denominator, because they may overestimate the reporting rate. Country-specific sales or exposure denominators were also not available in a sufficiently complete and comparable format. Consequently, the geographic distribution of reports could not be normalized by country and should be interpreted only as a descriptive map of reporting counts, not as a comparison of national incidence or reporting rates. Another limitation is that, although product-level information on RYR content and monacolin K content was available, the present analysis was not designed to correlate adverse-event reporting with the exact monacolin K content of individual products. The monacolin K content varied across products, but all included products were formulated to provide less than 3 mg/day of monacolin K according to the recommended daily dose, in line with the current European regulatory framework. This is relevant because the present findings describe the aggregate post-marketing safety profile of RYR-containing food supplements marketed below this regulatory threshold, rather than the comparative safety of individual formulations. Moreover, individual-level exposure could not be reconstructed with sufficient precision for all reports, because actual daily intake, duration and regularity of use, adherence to the recommended dose, and concomitant products or medications were variably documented. As a result, the present analysis cannot reliably assess dose–response relationships between exact monacolin K exposure and adverse-event reporting, nor can it compare safety profiles across individual products or formulation subgroups. The findings should therefore be interpreted as an aggregate post-marketing assessment of RYR-containing food supplements captured within this European nutrivigilance initiative, rather than as a product-specific or monacolin-dose-specific safety evaluation. Fourth, causality assessment in nutrivigilance remains probabilistic rather than definitive, especially in the presence of concomitant products, comorbidities, and incomplete rechallenge information. Finally, the present findings pertain to the surveillance framework and formulations captured within this initiative and should not be extrapolated indiscriminately to all RYR-containing products available on the market, given the well-known heterogeneity in composition and quality across formulations [18].

These limitations, however, coexist with important strengths. The study did not rely on unstructured anecdotal reporting alone, but on a common surveillance methodology supported by a dedicated case report form, active follow-up for missing or inconsistent information, medical expert review, formal causality assessment, and linkage to cumulative exposure estimates. This combination makes the dataset more clinically meaningful than simple tabulation of consumer complaints and brings it closer to a genuine post-marketing safety evaluation. Especially in a field where pre-marketing efficacy evidence is often more developed than post-marketing safety infrastructure, such methodological rigor represents a substantial advantage.

## 5. Conclusions

From a clinical and public health perspective, the present findings support a balanced interpretation of RYR-containing food supplements. They do not justify indiscriminate supplementation, nor do they suggest that RYR should be used as a substitute for indicated lipid-lowering pharmacotherapy in high-risk patients. Rather, they support the view that well-characterized RYR-containing products can be appropriately positioned within the framework of food supplements, provided that their use is supported by clear labelling, informed consumer choice, adherence to recommended conditions of use, and structured post-marketing nutrivigilance. In this perspective, the present data do not imply a need for pharmacy-controlled or prescription-based use, but instead reinforce the importance of responsible use within an evidence-based nutraceutical context. This is most coherent when RYR is framed within primordial prevention and population-level risk reduction, not as a shortcut around rigorous prevention, but as one possible component of it [8,18,26].

Further progress will depend on three complementary developments: better standardization of RYR-containing products [67], wider implementation of harmonized nutrivigilance procedures across Europe, and stronger integration between formulation-specific clinical evidence and post-marketing safety surveillance. These developments would improve regulatory confidence, enhance public trust, and help move the field from polarized debate toward a more mature, evidence-calibrated model of responsible food supplement use [8,50,53]. Future studies should also aim to incorporate more granular exposure denominators, product-level formulation data, including monacolin content when available, and standardized case-level follow-up, so that reporting rates can be interpreted with greater precision across products, countries, and consumer subgroups. At the same time, continued vigilance remains essential, particularly in the context of heterogeneous real-world use and evolving regulatory frameworks. From a public-health perspective, protecting citizens should not mean relying on generalized restriction alone, but investing in standardized, transparent, and publicly governed nutrivigilance and phytovigilance systems able to support responsible use, early signal detection, and evidence-based regulatory decisions.

## Figures and Tables

**Table 1:** Reporting and demographic characteristics of spontaneous adverse event reports associated with red yeast rice-containing food supplements.

Variable	Value
Age	
Information available, *n* (%)	93/148 (62.8)
Mean age, years (mean ± standard deviation)	65.3 ± 13.3
Age ≥ 70 years among reports with available age, *n*/N (%) ^1^	54/93 (58.1)
Sex	
Information available, *n* (%)	145/148 (98)
Female, *n*/N (%) ^1^	112/145 (77.2)
Male, *n*/N (%) ^1^	33/145 (22.8)

^1^ Percentages were calculated using the denominator shown in each row. Age-group percentages were calculated among reports with available age information (N = 93), not among all spontaneous reports (N = 148). Therefore, the proportion of individuals aged ≥ 70 years should be interpreted descriptively and with caution because age information was unavailable for 55 reports. Sex percentages were calculated among reports with available sex information (N = 145).

**Table 2:** Temporal profile, causality assessment, and severity of spontaneous adverse event reports.

Variable	*n* (%)
Time to onset	
<1 week	18 (12.2)
<1 month	5 (3.4)
>1 month	9 (6.1)
Unknown	116 (78.4)
WHO-UMC causality classification	
Certain	17 (11.5)
Probable/Likely	7 (4.7)
Possible	88 (59.5)
Unlikely	26 (17.6)
Unclassified	10 (6.8)
Severity grading	
Mild	126 (85.1)
Moderate	19 (12.8)
Severe	1 (0.7)
Serious	2 (1.4)

Percentages were calculated on the total number of reports. Time-to-onset information was unavailable in 116 of 148 reports (78.4%), leaving only 32 evaluable reports for temporal assessment. This high level of missingness represents a major limitation and may introduce reporting or recall bias. Therefore, onset categories should be interpreted as descriptive information from a limited subset of reports and should not be considered evidence of a reliable temporal pattern of adverse-event occurrence. Causality categories were assigned according to the WHO-UMC framework and should be interpreted as probabilistic assessments rather than definitive proof of a causal relationship. Severity grading reflects the information available in spontaneous reports, which may be incomplete. Abbreviations: WHO-UMC, World Health Organization–Uppsala Monitoring Centre.

**Table 3:** Exposure-based reporting rate estimates for red yeast rice-containing food supplements during the study period.

Variable	Primary Full-Adherence Exposure Equivalent	Hypothetical 50% Average Compliance Scenario
Cumulative tablets sold, *n*	328,759,726	328,759,726
Estimated exposed subject-periods, *n*	608,815	1,217,630
Exposure-based reporting rate of all AE reports	0.024%	0.012%
Estimated-based reporting rate of SAE reports	0.00033%	0.00016%

Exposure estimates were derived from cumulative manufacturing and sales data available up to 31 May 2024 and converted into exposed subject-periods on the basis of the recommended daily dosage. The primary estimate assumes continuous use at the recommended daily dose over the study period. The hypothetical 50% compliance scenario was calculated by doubling the estimated exposure denominator, because the same number of sold units would correspond to a larger number of exposed subject-periods under reduced average compliance. These scenarios should be interpreted as approximate exposure-based reporting rates rather than true epidemiological incidence rates. They were intended to provide a plausible range of estimates, as individual-level compliance data were not available and real-world use is likely to be intermittent. Abbreviations: AE, adverse event; SAE, serious adverse event.

## Data Availability

The data presented in this study are available from EHPM upon reasonable request. The data are not publicly available because of legal and privacy constraints.

## Supplementary Materials

The following supporting information can be downloaded at: https://www.mdpi.com/article/10.3390/nu18142302/s1, File S1. Case Report Form for Nutrivigilance Data Collection.

## Abbreviations

The following abbreviations are used in this manuscript:

AE	Adverse events
ASCVD	Atherosclerotic cardiovascular disease
CRF	Case report form
EAS	European Atherosclerosis Society
EFSA	European Food Safety Authority
EHPM	European Federation of Associations of Health Product Manufacturers
ESC	European Society of Cardiology
EU	European Union
hsCRP	High-sensitivity C-reactive protein
LDL-C	Low-density lipoprotein cholesterol
NV Officer	Nutrivigilance Officer
PRISMA	Preferred Reporting Items for Systematic Reviews and Meta-Analyses
RYR	Red yeast rice
WHO	World Health Organization
WHO-UMC	World Health Organization–Uppsala Monitoring Centre
