# Supplementary material for: Post-Marketing Nutrivigilance of Red Yeast Rice-Containing Food Supplements: Spontaneous Adverse Event Reports and Exposure-Based Reporting Rate Estimates
Source: Nutrients. 2026 Jul 14;18(14):2302. doi: 10.3390/nu18142302 (PMC13414495; doi:10.3390/nu18142302)
Supplement: Supplementary file 1 [file nutrients-18-02302-s001.zip › nutrients-4360448-supplementary.pdf]

Case Report Form

To be compiled by the Company/Company Vigilance department:

Unique number:	
Case Received on (Initial Received Date):	(DD-MM-YYYY)
Is this a Follow-up?	Yes ☐ No ☐
If this is a Follow-up, date of this Follow-up received:	(DD-MM-YYYY)

ATTACH to the report, once completed, a copy of the PRODUCT CARTON including ingredients, warnings, mode of use.

---

1. End-User Details

Initials:	Sex: Male ☐ Female ☐	Birth date/age:	Country of Incidence:
Pregnant (if applicable): Yes ☐ / No ☐ If Yes, date of last menstrual period:			

**2. Suspect Product(S) Names**	**Start - End Use Dates** (duration if dates are unknown)	**Motivation of use** (can be different from the Product Label)	**Number of intakes** (total, and per day/week)
1.<br><br>Lot n.:<br>Best Before End Date:			
2.<br><br>Lot n.:<br>Best Before End Date:			

Add rows if needed.

3. Event	Onset date of event	End date of event (write duration if dates are unknown)	Outcome	
1.			Recovered	☐
			Recovering	☐
			Not recovered	☐
			Recovered with sequelae	☐
			Unknown	☐
2.			Recovered	☐
			Recovering	☐
			Not recovered	☐
			Recovered with sequelae	☐
			Unknown	☐

4. Description of what happened including consequences due to adverse event, if any

____

____

____

____

5. Relevant medical history and concomitant medication/other[1]

(Allergies, concurrent illnesses or treatments, relevant past illnesses or treatments, similar products used, etc.)

____

____

____

____

____

[1] *Mention other medications or food supplements or botanicals or foods or other products such as homoeopathic, used in combination or within nearly 2 months from the use of the product. If not enough rows, please enclose full list.*

6. Dechallenge/Rechallenge	
Was the suspect Product discontinued?<br>Yes ☐ No ☐<br>If yes, did the event abate?<br>Yes ☐ No ☐ Not Applicable ☐ Unknown ☐	Was the Product introduced/used again?<br>Yes ☐ No ☐<br>If yes, did the event reoccur?<br>Yes ☐ No ☐ Not Applicable ☐ Unknown ☐

7. The event/events has/have caused one of the following situations?

☐ Hospitalisation or prolongation of existing hospitalisation (at least 1 calendar day)

☐ persistent or significant disability or incapacity

☐ a congenital anomaly/birth defect

☐ life-threatening

☐ death

☐ none of the above, but physician/pharmacist/HCP considered the event serious

☐ none of the above. The product could have caused any of the above, but it was not taken (i.e. contaminated tablet, presence of an external body)

8. Have you reported this case to your National Competent Authority?

Yes ☐ Date: ……… Reference number. (if known): ……

No ☐

9. Reporter's Details	
Name, Surname:	
Specification:	Doctor ☐ Nurse ☐ Pharmacist ☐ Nutritionist ☐ Consumer ☐<br>Other (please, specify) ☐
Address	
Tel no:	
Email:	

**10. Do you/Does the reporter provide consent to be contacted for further information?**
Yes ☐ No ☐

**11. If end-user reports:**
Was there any contact with HCP regarding the AE(s)? Yes ☐ No ☐
Do you/Does the reporter provide consent for their HCP to be contacted by the Company for further information?<br>☐ Yes (HCP details for contact: ....)<br>☐ No<br>☐ N/A as Reporter is HCP

Data protection statement to be added by the Company
